# Chemo-RaST with bortezomib inhibits multiple myeloma relapse

**DOI:** 10.7150/thno.111508

**Published:** 2025-09-08

**Authors:** Anchal Ghai, Alexander Zheleznyak, Christopher Egbulefu, Nicole Blasi, Kvar Black, Rui Tang, Matthew L Cooper, Kiran Vij, Ravi Vij, John DiPersio, Monica Shokeen, Samuel Achilefu

**Affiliations:** 1 Department of Radiology, Washington University School of Medicine, St. Louis, Missouri, USA.; 2 Department of Biomedical Engineering, UT Southwestern Medical Center, Dallas, Texas, USA.; 3 Department of Medicine, Washington University School of Medicine, Missouri, USA.; 4 Department of Pathology and Immunology, Washington University School of Medicine, Missouri, USA.; 5 Department of Biomedical Engineering, Washington University in St. Louis, Missouri, USA.; 6 Department of Biochemistry and Molecular Biophysics, Washington University School of Medicine, Missouri, USA.

**Keywords:** myeloma, Radionuclide-stimulated dynamic therapy (RaST), chemo-RaST, bortezomib, relapse, therapy resistance

## Abstract

**Rationale:** Multiple myeloma (MM) is a hematological malignancy with a high relapse rate that ultimately leads to patient mortality. Current therapies often fail to achieve sustained remission due to adaptation of clonally heterogeneous tumor populations. We hypothesized that Chemo-RaST, a therapeutic strategy combining bortezomib with ⁸⁹Zr-daratumumab-mediated radionuclide dynamic therapy (RaST), would synergize photophysical generation of cytotoxic reactive oxygen species (ROS) with mitochondrial ROS induction to block clonal adaptation and prevent MM relapse.

**Methods:** We evaluated chemo-RaST in MM.1S-luc subcutaneous and disseminated MM mouse models. RaST consisted of zirconium-89 (⁸⁹Zr)-labeled daratumumab to target MM cells and continuously activate orthogonally delivered titanium dioxide-transferrin-titanocene (TiO₂-Tf-TC) nanoparticles for sustained cytotoxic ROS production. Bortezomib, a proteasome inhibitor, was administered in parallel to amplify mitochondrial ROS. Therapeutic efficacy was evaluated using bioluminescence imaging (BLI), positron emission tomography (PET), and histopathology.

**Results:** *In vitro*, RaST reduced MM.1S-luc cell viability to 48.4 ± 2.0% versus untreated controls (98.7 ± 1.5%), TiO₂-Tf-TC nanoparticles alone (96.6 ± 0.8%), or ⁸⁹Zr-daratumumab alone (91.3 ± 3.4%). *In vivo*, RaST suppressed tumor progression, but relapse occurred. In contrast, chemo-RaST achieved complete tumor regression in 60% of disseminated MM models and significantly extended progression-free survival. Histopathology confirmed elimination of CD138-positive MM cells and restoration of normal hematopoiesis in Chemo-RaST cohorts.

**Conclusions:** Tracer doses of long-lived ⁸⁹Zr for sustained photosensitizer activation, combined with subtherapeutic bortezomib, represent a clinically translatable strategy to limit off-target toxicity, prevent relapse, and overcome therapy resistance in multiple myeloma.

## Introduction

Multiple myeloma (MM), the second most common hematological malignancy, originates in bone marrow and primarily affects plasma cells 1. As the disease progresses, it often spreads to various skeletal and soft tissues, including the spine, kidneys, and liver 2. Classic symptoms of MM include bone pain and fractures, elevated blood calcium levels, and severe anemia. The high clonal heterogeneity complicates treatment planning and response, making durable remission challenging. Consequently, the standard of care typically combines chemotherapeutics, pathway inhibitors, and biologics to disrupt cancer's multiple survival mechanisms 3, 4. Despite improved survival, MM remains incurable mainly due to resistant subclones that contribute to frequent relapse 4-6, underscoring the need for synergistic strategies that eradicate MM effectively.

Radionuclide-stimulated dynamic therapy (RaST), also known as Cerenkov Radiation-Induced Therapy, is one of such strategies 7, 8. During RaST, Cerenkov-emitting radionuclides activate photosensitizers to generate cytotoxic ROS 7, 9-11. To minimize off-target toxicity and selectively kill tumor cells, both components are delivered orthogonally to the same cells 12. Clinically approved positron emitters, including ¹⁸F, ⁶⁴Cu, ⁶⁸Ga, ⁸⁹Zr, ⁹⁰Y, and ¹²⁴I, serve as sources of Cerenkov radiation for RaST 13, 14 with demonstrated success in inhibiting solid tumor progression in preclinical models 15. Among these, ⁸⁹Zr is advantageous owing to its long half-life (78.4 h) and suitable positron emission characteristics (β_max_ = 897 keV) 16. Preclinical investigations have shown that radiolabeled analogs of the FDA-approved therapeutic monoclonal antibody, daratumumab, can be used to image and quantify CD38 expression *in vivo*, providing valuable insights into tumor size and location 4, 17. The long physical half-life of ^89^Zr aligns with the extended biological half-life of monoclonal antibodies (~14 days in humans and ~7-8 days in mice), making ⁸⁹Zr-daratumumab an ideal Cerenkov source for optimizing RaST and monitoring responses in CD38-overexpressing myeloma cells.

We hypothesized that combining photophysical stimulation of cytotoxic ROS with drug-induced mitochondrial ROS generation (chemo-RaST) would inhibit clonal adaptation and prevent MM relapse. We selected the regenerative photocatalyst titanium dioxide (TiO₂) nanoparticles 18 and ^89^Zr to sustain cytotoxic ROS generation over extended periods. Orthogonal delivery was achieved by coating TiO₂ with apo-transferrin (Tf) and radiolabeling daratumumab to target transferrin receptors (CD71) and CD38, respectively, which are overexpressed on rapidly proliferating myeloma cells 19. Given MM's clonal heterogeneity, we combined RaST with the clinically approved proteasome inhibitor, bortezomib 20, 21, to provide complementary mitochondrial ROS. Our results demonstrate, to our knowledge, the first evidence that chemo-RaST achieves prolonged progression-free survival in preclinical MM models.

## Materials and Methods

### Materials

Daratumumab, an FDA-approved humanized monoclonal antibody against CD38, was provided by the Siteman Cancer Center pharmacy at Washington University in St. Louis for research purposes. Deferoxamine-p-benzyl-isothiocyanate (DFO-Bz-NCS) was purchased from Macrocyclics, Inc. TiO_2_ nanoparticles and other chemicals were obtained from Sigma-Aldrich unless otherwise noted. Transferrin-Alexa 680 dye conjugate was purchased from Thermo Fisher Scientific (USA). Bortezomib was obtained from Sigma-Aldrich. All solutions were prepared in ultrapure water (18 MΩ·cm, Millipore). Instruments were calibrated and maintained per the manufacturer's instructions.

### Synthesis and characterization of TiO_2_-Tf-TC nanoparticles

Titanium dioxide-transferrin-titanocene (TiO_2_-Tf-TC) nanoparticles were synthesized as described previously 7. Briefly, 25 nm anatase TiO_2_ nanoparticles (Sigma Aldrich Co., St. Louis, USA) were suspended in deionized water (10 mg/mL stock). Stock was mixed vigorously with Tf in phosphate-buffered saline (PBS) at a 1:3 TiO_2_:Tf mass ratio and dispersed by probe sonication (3 W, 60 s; Cole-Parmer Ultrasonic Processor GE 130PB (Vernon Hills, IL)). The mixture was immediately filtered through a 0.22 µm polyethersulfone syringe filter (VWR Scientific, Batavia, IL), then 2% v/v of 12 mM titanocene (TC; Alfa Aesar, Tewksbury, MA) in DMSO was added, yielding TiO_2_-Tf-TC nanoparticles. Size was measured by dynamic light scattering (DLS; Malvern Zetasizer Nano ZS). Transmission electron microscopy (TEM) images were acquired on an FEI Tecnai Spirit (Hillsboro, Oregon, USA) at 80 keV. Grids were prepared by depositing 2 µL of sample on formvar/carbon grids (200 mesh, Ted Pella Inc.) and soaking up excess droplets with filter paper. Freshly formulated nanoparticles were used within 4 h of preparation.

### ^89^Zr production

Radioactive materials were used under Washington University's Nuclear Regulatory Commission license. ⁸⁹Zr was produced on a CS-15 cyclotron at the School of Medicine's Cyclotron Facility. Activity was measured with a Capintec CRC-15R dose calibrator (Ramsey, NJ, USA). Radiolabeling efficiency was monitored by instant thin-layer chromatography (iTLC) using iTLC-SG paper (Agilent Technologies, CA, USA) and a Scan RAM radio-TLC scanner (LabLogic, Brandon, FL, USA). pH was measured with pH paper strips (EMD Chemicals, Inc., Gibbstown, NJ, USA).

### Synthesis and radiolabeling of daratumumab with ^89^Zr

Daratumumab was conjugated to DFO-Bz-NCS (10-15-fold molar excess), and the conjugate was radiolabeled with neutralized ⁸⁹Zr as described 4. Briefly, ⁸⁹Zr-oxalate (pH ≤ 2) was adjusted to pH 6.8-7.2 by adding 1 M HEPES (pH 7) followed by stepwise 1 M NaOH. Daratumumab concentration was determined by BCA assay (A@570-580 nm). Zeba spin desalting columns (40 kDa cut-off; Thermo Fisher, Rockford, IL, USA) were used to remove excess chelator and free ⁸⁹Zr using 1 M HEPES as exchange buffer. Radiochemical purity (RCP) of ⁸⁹Zr-daratumumab was confirmed by radio-TLC using 50 mM DTPA as the mobile phase.

### Cell lines

The human MM.1S cell line expressing luciferase (MM.1S-luc) was provided by Dr. John F. DiPersio (Washington University School of Medicine). Cells were cultured in RPMI-1640 (Thermo Fisher) with 10% fetal bovine serum (Gibco) and 1% streptomycin (Corning, Manassas, USA) at 37 °C, 5% CO₂.

### Flow cytometry

MM.1S cells (1 × 10⁶) were resuspended in staining buffer (PBS with 0.5% BSA and 2 mM EDTA) and incubated for 30 min at 4 °C with fluorochrome-labeled monoclonal antibody anti-human CD71-PE-Cy7 (clone CY1G4, BioLegend) or isotype control (MOPC-173, BioLegend). Data were acquired on a Gallios cytometer and analyzed with FlowJo v10 software.

### *In vitro* cell imaging

MM.1S-GFP cells were incubated for 4-24 h at 37 °C with Tf-Alexa 680 (final 180 µg/mL) or TiO₂-Tf-Alexa 680 (100 µL per 1 mL). Cells were washed twice with PBS and plated on MatTek glass-bottom dishes. Olympus FV1000 confocal microscope (60× objective; 633 nm excitation; 655-755 nm emission) was used for fluorescence and bright-field imaging. Images were processed with Fluoview FV10-ASW software.

### TiO_2_-Tf-TC nanoparticle cell internalization assay

MM.1S cells were plated in 24-well TPP tissue culture plates (Midwest Scientific, St. Louis, MO) at 2 × 10⁶ cells/well in 1 mL RPMI with 10% FBS, 10,000 U/mL penicillin, and 10,000 µg/mL streptomycin. Immediately after seeding, 100 µL of TiO₂-Tf-TC suspension containing 50 µg TiO₂ was added per well and mixed. Plates were incubated at 37 °C and 5% CO_2_ humidified atmosphere in a NUAIR IR AUTOFLOW CO_2_ water-jacketed incubator (Plymouth, MN) for 24 h. Medium containing unbound nanoparticles was removed. Membrane-bound fraction was collected by incubating cells with 0.1 M sodium citrate (pH 2) for 5 min, followed by centrifugation, and an additional PBS wash (1 mL). These washes were pooled as the membrane-bound fraction. The remaining cell pellet was collected as the internalized fraction.

For titanium (Ti) quantification, samples were digested in 2 mL HNO₃ (70%, trace metal, Sigma-Aldrich), 1 mL HCl (37%), and 1 mL H₂O₂ (30%) in sealed vessels (Mars 6 Microwave Digestion System; CEM Corporation); ramp to 200 °C over a period of 20 min, and maintained for 20 min. Samples were cooled and diluted to 5 mL with 18 MΩ water. Ti concentration was measured using a PerkinElmer Optima 7300DV ICP-OES with continuous internal standard (200 µg/L ⁴⁵Sc). Calibration standards were 1, 10, 100, and 1000 µg/L. Ti concentration in the cells was calculated by multiplying the measured Ti concentration by the dilution factor and the known sample volume, then dividing by the tissue weight.

### *In vitro* RaST

Cytotoxic effects of RaST with ⁸⁹Zr-daratumumab and TiO_2_-Tf-TC nanoparticles were evaluated in 24-well plates (TPP, Midwest Scientific, St. Louis, MO, USA). Each well contained 4 × 10⁶ MM.1S cells (no reporter) in 1 mL complete growth medium. Treatments were: TiO₂-Tf-TC (50 µg/well), ⁸⁹Zr-daratumumab (0.0037 MBq/well), or RaST, i.e., ⁸⁹Zr-daratumumab (0.0037 MBq/well) in combination with TiO_2_-Tf-TC nanoparticles (50 µg/well). Cells were incubated for 72 h at 37 °C, 5% CO₂. Medium was replaced with 500 µL PBS, and viability was measured using CellTiter 96 Aqueous One Solution Cell Proliferation Assay(Promega, Madison, WI, USA). Emitted fluorescence was captured on a BioTek NeO_2_ hybrid multimode reader.

### MM.1S-luc subcutaneous and disseminated MM mouse models

All animal studies followed the guidelines of the Washington University and UT Southwestern Animal Studies Committees. Female Fox Chase SCID Beige (FCSB) mice, 4-6 weeks old (Charles River Laboratories, USA), were acclimatized for 1 week with ad libitum food and water. Euthanasia was caused by cervical dislocation under 5% isoflurane anesthesia. Reporter expression was verified by *in vitro* BLI (1 × 10⁵ cells/well, black 96-well plate). For subcutaneous models, MM.1S-luc cells (1 × 10⁶ cells/mouse) in PBS were injected into the right flank, and tumors were allowed to grow for 10-14 days. For disseminated disease, MM.1S-luc cells (1 × 10⁶/100 µL) in PBS were injected via the tail vein.

### *In vivo* RaST and chemo-RaST in subcutaneous tumor-bearing mice

Study design and RaST regimen are shown in Figure 3. Subcutaneous MM.1S-luc tumor-bearing mice were assigned to untreated, TiO-Tf-TC nanoparticles only (100 µg/100 µL), bortezomib only (1 mg/kg), ⁸⁹Zr-daratumumab only (1-1.2 MBq/100 µL), or RaST (the same dose of ⁸⁹Zr-daratumumab was used, followed 3 days later by TiO₂-Tf-TC). The radiotracer was injected on days 21 and 35 in the ⁸⁹Zr-daratumumab and RaST groups, followed 3 days later (days 24 and 38) by TiO₂-Tf-TC nanoparticles. This timing allowed for selective tumor uptake of ⁸⁹Zr-daratumumab prior to nanoparticle delivery. Mice were monitored weekly by BLI. For chemo-RaST, bortezomib (1 mg/kg, administered intraperitoneally) was given twice weekly, starting simultaneously with RaST initiation.

### *In vivo* RaST and chemo-RaST in the disseminated myeloma model

Disseminated myeloma models were used to simulate systemic MM. Disease burden was monitored weekly by BLI, and treatment was initiated when total flux reached 1 × 10⁶. Mice were divided into six groups: untreated; TiO-Tf-TC only (100 µg/100 µL); ⁸⁹Zr-daratumumab only (1-1.2 MBq/100 µL); bortezomib only (1 mg/kg); RaST (⁸⁹Zr-daratumumab followed 3 days later by TiO-Tf-TC); and chemo-RaST. Doses for each RaST or chemo-RaST component were kept consistent in the combination therapy. Bortezomib was administered at 1 mg/kg twice weekly via intraperitoneal. Response and disease progression were assessed by BLI.

### Bioluminescent imaging

Tumor progression in vivo was monitored weekly by BLI using IVIS Lumina system (Living Image 3.2; 1-300 s exposures; binning 2-8; field of view 12.5 cm; PerkinElmer, Waltham, MA, USA). Mice received an intraperitoneal injection of 150 mg/kg D-luciferin in PBS and were imaged 10 minutes later under isoflurane anesthesia (2% in O₂). For subcutaneous or disseminated models, regions of interest (ROIs) were drawn around tumors or over the dorsal/ventral sides. Total flux (photons/sec) was measured using Living Image 2.6 and normalized to average radiance (photons/sec/cm²/sr). The first treatment dose was administered when total flux reached 1 × 10⁶.

### *In vivo* PET imaging in subcutaneous tumor-bearing mice

Mice in the ⁸⁹Zr-daratumumab and RaST groups received ⁸⁹Zr-daratumumab (1-1.2 MBq) on days 21 and 35. PET/CT imaging was performed 3 days post-injection. The day 24 time point served as baseline (pre-therapy) and preceded TiO-Tf-TC injection in the RaST cohort. Post-therapy imaging was performed on day 38. Mice were anesthetized with isoflurane (1-2%) and imaged on a small-animal PET/CT scanner. Thirty-minute static scans were co-registered and reconstructed using Inveon Research Workstation (IRW) software. ROIs were drawn around the tumors from PET images using CT anatomical guidelines, and the SUV_mean_ was calculated from these ROIs.

### Histology

Hematoxylin and eosin (H&E) staining and CD138 immunohistochemistry (IHC) were performed on 5 µm sections from decalcified, formalin-fixed, paraffin-embedded leg bone tissue from the MM.1S-luc mice. A prediluted CD138/syndecan-1 (B-A38) monoclonal antibody on the VENTANA BenchMark platform was utilized for CD138 IHC. A single pathologist, blinded to treatment, reviewed H&E and CD138 slides from untreated and treated cohorts. Representative images were captured at original magnification ×100 (10× objective) using an OLYMPUS BX51 microscope.

### Statistical analysis

Statistical analyses were performed using GraphPad Prism 8.0 (GraphPad Software, Inc., La Jolla, CA, USA). Data are expressed as mean ± standard deviation unless otherwise noted. p < 0.05 was considered statistically significant.

## Results

### Synthesis and characterization of RaST components

Our experimental design employs two strategies to selectively deliver both the Cerenkov-emitting radiopharmaceutical and photosensitizer to MM cells at non-cytotoxic doses. Previous studies have demonstrated that DFO forms stable chelates with ^89^Zr 24. Following established protocols 4, we conjugated daratumumab with DFO and radiolabeled the daratumumab-DFO conjugate using ^89^Zr-oxalate. Radiochemical purity exceeded 99%, and the labeling efficiency was confirmed by radio-TLC (Figure S1).

**Figure 1:**
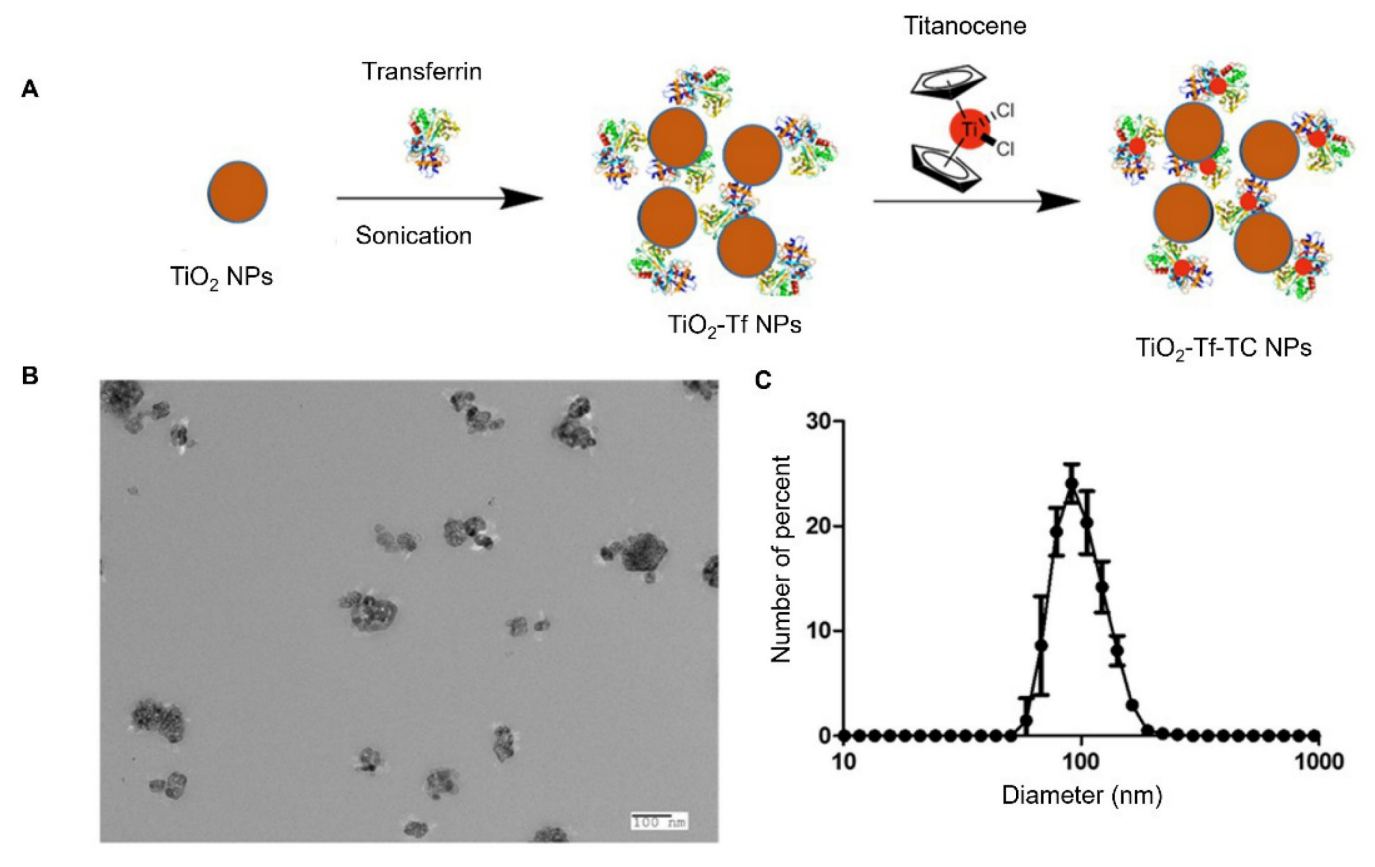
** Synthesis and characterization of TiO**_2_**-Tf-TC nanoparticles**. (A) Synthetic schematic of TiO_2_-Tf-TC nanoparticles. (B) Representative transmission electron microscope (TEM) image of TiO_2_-Tf-TC nanoparticles. (C) Dynamic light scattering (DLS) of TiO_2_-Tf-TC nanoparticles.

Incorporating TC, a biocompatible photoinitiator, into TiO₂ nanoparticles has been shown to enhance the RaST effect 7. TC forms stable chelates with apo-transferrin, which binds to anatase-phase TiO₂ nanoparticles 7. Functionalizing the metal oxide surface with Tf enhanced nanoparticle dispersion and facilitated loading with TC, enabling targeted delivery to transferrin receptors overexpressed on MM cells (Figure 1A). TEM confirmed that the synthesized TiO₂-Tf-TC nanoparticles were approximately 100 nm in size (Figure 1B), consistent with the 105 nm hydrodynamic diameter measured by DLS (Figure 1C). Nanoparticle suspensions were well-dispersed and exhibited a low polydispersity index of 0.1, indicating uniform particle size distribution.

### Internalization of TiO_2_-Tf nanoparticles and RaST in MM cells

**Figure 2:**
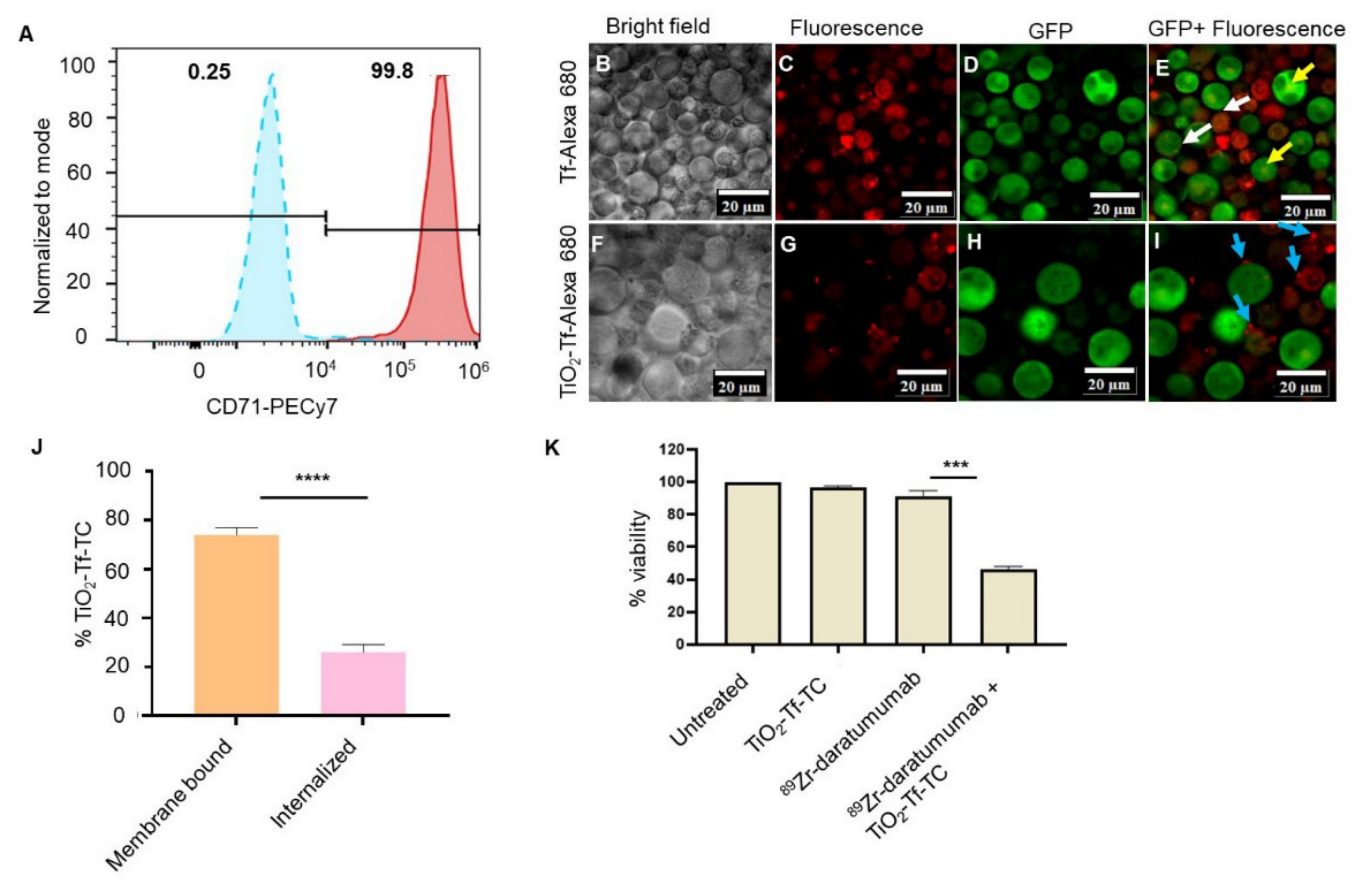
** Expression of targeted biomarkers and cellular activity of NPs.** (A) Flow cytometry of MM.1S human MM cells for transferrin receptor expression (B-I) Confocal fluorescence imaging of MM.1S cells after incubation with Transferrin-Alexa 680 (Tf-Alexa 680) and TiO_2_-Transferrin-Alexa 680 (TiO_2_-Tf-Alexa 680) (J) Quantification of TiO_2_-Tf-TC internalization in MM.1S MM cells by ICP-MS after 24-hour incubation, (K) MTS assay after RaST along with controls. **** p-value <0.0001, ** p-value = 0.007.

The MM.1S cell line is commonly used for preclinical MM studies 25. These cells were isolated from the peripheral blood of a patient with MM who was resistant to corticosteroids. MM.1S cells overexpress CD38, enabling targeted delivery of daratumumab 26. However, using daratumumab to deliver both photosensitizer and radionuclide would create competition for CD38 and reduce receptor-mediated internalization. To circumvent this, we evaluated orthogonal delivery of nanophotosensitizers via transferrin receptors (CD71). Flow cytometry showed high CD71 expression (>99%) on MM.1S cells versus <0.5% in the isotype control (Figure 2A), enabling dual targeting of CD71 and CD38 on the same cell. The MM.1S cells used *in vitro* and *in vivo* were stably transfected with luciferase.

Confocal imaging after 24 h incubation with transferrin protein-Alexa 680 (Tf-Alexa 680) revealed punctate vesicular uptake at the plasma membrane (Figure 2B-E) and diffuse subcellular fluorescence in mononuclear cells (white arrows), with centralized localization in multinucleated cells (yellow arrows). Similarly, TiO₂-Tf-Alexa 680 nanoparticles showed strong membrane-associated fluorescence (blue arrows) in most cells, with low uptake in highly aggressive multinucleated cells (Figure 2F-I). Because Tf-Alexa 680 can dissociate from nanoparticles over time in cells, potentially skewing uptake analysis, we quantified Ti⁴⁺ by ICP-MS after overnight incubation of 2 × 10⁵ MM.1S cells/well with 50 µg TiO₂-Tf-TC. Approximately 75% of cellular Ti⁴⁺ was membrane-bound and 25% internalized (Figure 2J).

We next established non-cytotoxic doses of ⁸⁹Zr-daratumumab (0.0037 MBq) and TiO₂-Tf-TC nanoparticles (50 µg) to assess RaST-mediated cell death by MTS assay. Under these conditions, untreated (98.7 ± 1.5%), nanoparticle-treated (96.6 ± 0.8%), and ⁸⁹Zr-daratumumab-treated cells (91.3 ± 3.4%) showed no significant loss of viability (Figure 2K). In contrast, RaST (⁸⁹Zr-daratumumab followed by TiO₂-Tf-TC) reduced viability to 48.4 ± 2.0%, demonstrating substantial cytotoxicity.

### RaST and chemo-RaST in the MM.1S-luc subcutaneous myeloma model

Subcutaneous and disseminated MM models were used to evaluate whether RaST can prevent relapse. Although MM is primarily hematologic with multifocal marrow lesions, soft-tissue extramedullary myelomas are common 27 and resemble solid tumors modeled by subcutaneous implants.

Since positrons from ^89^Zr can travel approximately 2 mm before annihilation, we expected activation of nanophotosensitizers bound to the plasma membrane and those internalized. However, effective RaST depends on intracellular production of cytotoxic ROS. Microscopy shows heterogeneous uptake, particularly limited or peripheral accumulation in multinucleated cells. This indicates that the more aggressive subpopulation may evade adequate intracellular activation, reducing RaST effectiveness and possibly leading to post-treatment relapse. One strategy to address this is to combine RaST with small drug molecules that can internalize into these cells. Bortezomib, a small molecule proteasome inhibitor widely used to treat MM, triggers cellular stress and apoptosis 28, which can stimulate mitochondrial ROS production 29. Bortezomib's small size enables diffusion into the multinuclear MM cells, enabling multidimensional ROS generation from mitochondrial (bortezomib) and photophysical (RaST) sources in tumors to eradicate the different myeloma clones before they become resistant.

**Figure 3:**
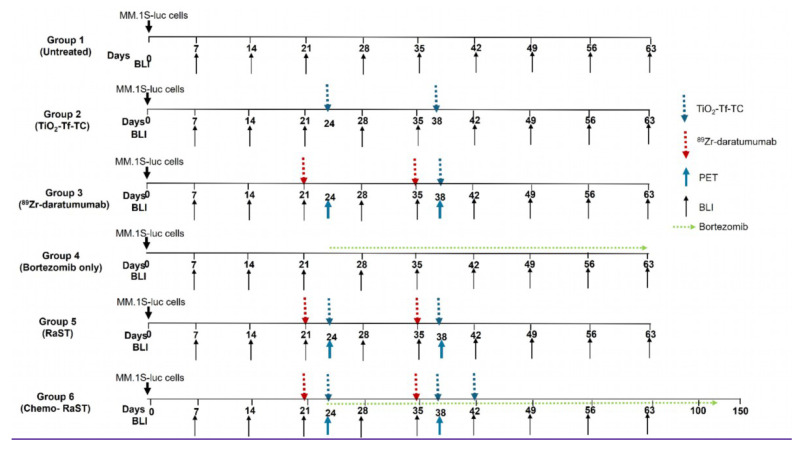
** Schematic representation of RaST regimen.** Fox Chase SCID Beige mice were injected subcutaneously (sq) with human myeloma cell lines, MM.1S-luc cells. Tumor progression was monitored weekly by BLI. Mice were divided into the following groups: Group 1 - MM.1S-luc tumor-bearing mice with no treatment, Group 2 - MM.1S-luc tumor-bearing mice treated with TiO_2_-Tf-TC nanoparticles only on days 24 and 38, Group 3 - myeloma tumor-bearing mice injected with ^89^Zr-daratumumab on days 21 and 35, Group 4 - mice treated with bortezomib only (1mg/kg, twice a week) starting on day 24, group 5 - mice with myeloma tumors treated with ^89^Zr-daratumumab followed by TiO_2_-Tf-TC nanoparticle treatment at 3 days later, and Group 6 - mice treated in combination with RaST and bortezomib. The bortezomib treatment was started along with RaST. Mice in groups 3 & 4 were imaged with ^89^Zr-daratumumab on days 24 (pre-therapy) and 38 (post-therapy) to evaluate therapy response.

Weekly BLI was used to monitor tumor progression and responses to TiO₂-Tf-TC nanoparticles only (100 µg/100 µL), bortezomib only (1 mg/kg), ^89^Zr-daratumumab only (1.2 MBq/100 µL), or RaST (1.2 MBq/100 µL ^89^Zr-daratumumab, followed by 100 µg/100 µL TiO₂-Tf-TC nanoparticles; Figure 3). Because daratumumab circulates for approximately 7-8 days in mice and ⁸⁹Zr has a 78.4 h half-life, the radiotracer was injected first to allow clearance from liver and non-tumor tissues and to maximize tumor accumulation. TiO₂-Tf-TC nanoparticles were administered 3 days later. Here, ⁸⁹Zr served as both a Cerenkov source and a PET radionuclide. For chemo-RaST, bortezomib (1 mg/kg, intraperitoneally) was given twice weekly, starting concurrently with RaST initiation.

**Figure 4:**
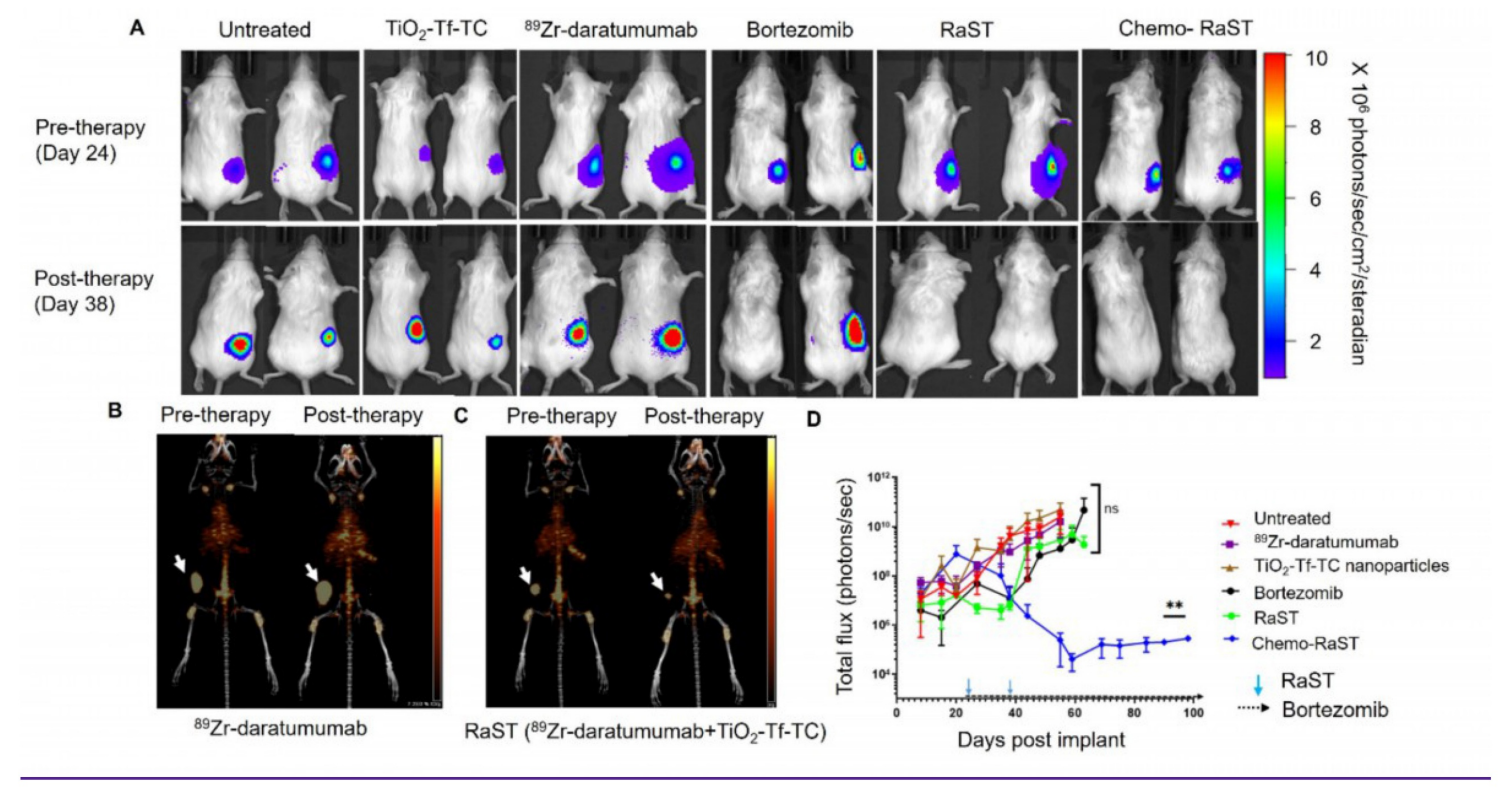
***In vivo* Chemo-RaST in subcutaneous MM model.** (A) Representative BLI images for MM.1S-luc subcutaneous tumor-bearing animals showing tumor progression in different treatment groups pre- (day 24)- and post-therapy (day 38). Representative MIP PET/CT images of ^89^Zr-daratumumab in (B) myeloma tumor-bearing mice treated with ^89^Zr-daratumumab alone acquired on days 24 and 38 (C) myeloma tumor-bearing mice treated with RaST on days 24 (pre-therapy) and 38 (post-therapy). Tumors = white arrows. (D) BLI was quantified for MM.1S-luc subcutaneous tumor-bearing animals of each group plotted and is shown as a function of the number of days after transplantation. **p-value = 0.0025. The sample size for different groups - untreated (n = 3), TiO_2_-Tf-TC (n = 4), ^89^Zr-daratumumab (n = 4), Bortezomib (n = 3), RaST (n = 4), Chemo-RaST (n = 3).

BLI showed tumor progression in untreated mice and in those receiving TiO₂-Tf-TC nanoparticles alone, ⁸⁹Zr-daratumumab alone, or bortezomib (Figure 4A; Figure S2). One bortezomib-treated animal with a low initial tumor mass did not develop BLI-visible signals within 10 days post-treatment. In contrast, RaST suppressed tumor growth across all treated mice. To better quantify response and mitigate light attenuation in tissues, we leveraged ⁸⁹Zr PET. A tracer dose of ⁸⁹Zr-daratumumab (1 MBq) was administered to the RaST and ⁸⁹Zr-daratumumab-only groups on day 21, and PET/CT was acquired on day 24, before initiating RaST. Follow-up PET/CT on day 38 followed an additional ⁸⁹Zr-daratumumab dose on day 35. PET/CT showed significant radiotracer retention in subcutaneous tumors (Figure 4B,C). In the ⁸⁹Zr-daratumumab group, tumor size and SUV _mean_ increased (2.6 ± 0.6 to 3.0 ± 1.0). RaST reduced tumor size and decreased SUV_mean_ from 2.8 ± 0.84 to 1.9 ± 0.27 two weeks after therapy. Residual tumors in the RaST group indicated potential for relapse.

As anticipated, the initial benefits of bortezomib or RaST diminished, with progressive disease re-emerging after day 40 (Figure 4D, Figure S3). This observation aligns with clinical outcomes, where relapses following an initial response are common in MM patients 30. Our rescue efforts with bortezomib (1 mg/kg, twice weekly on days 51-60; Figure S3A) were unsuccessful, as the tumor burden remained high, and all tumors reached the approved study endpoints. We postulated that initiating bortezomib concurrently with RaST could limit the selection of clones reprogrammed to resist ROS-mediated cell death 29, 31, 32. Consistent with this, chemo-RaST, followed by maintenance therapy, sustained therapeutic effects and prevented relapse (Figure 4A,D). This result highlights both the challenge of treating relapsed disease and the value of early combination intervention.

### *In vivo* chemo-RaST in the disseminated tumor model

**Figure 5:**
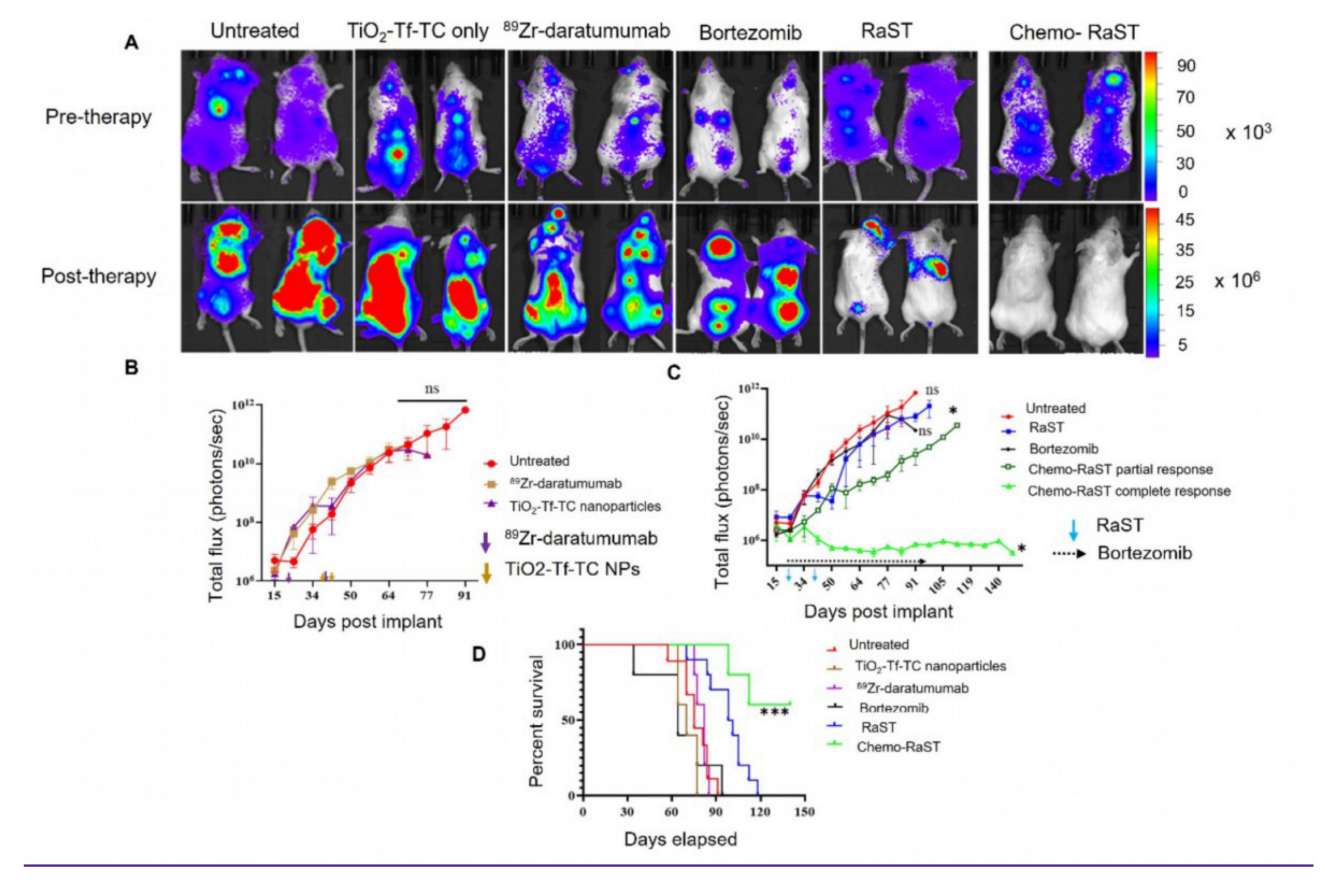
***In vivo* Chemo-RaST in disseminated MM model.** (A) Representative BLI images for MM.1S-luc disseminated tumor-bearing mice showing tumor progression in different treatment groups pre- (day 21)- and post- (day 50) therapy. Complete animal images are shown in Supplementary Fig S4 (B) Comparison of BLI signal for disseminated MM.1S-luc tumor-bearing mice: untreated cohort (n = 9) and mice treated with ^89^Zr-daratumumab alone (n = 5) and TiO_2_-Tf-TC nanoparticles alone (n = 5) (C) BLI signal was quantified and plotted as a function of number of days post-implantation of tumors in the disseminated MM.1S-luc tumor-bearing mice: untreated (n = 9), bortezomib only (n = 5), RaST (n = 10), and chemo-RaST (n = 5) groups. *p-value < 0.05 (D) Kaplan-Meier survival curve analysis of mice with disseminated myeloma disease with different treatments. ***p-value <0.0001.

MM is a multifocal and disseminated disease, necessitating a model that accurately mirrors its clinical presentation. To achieve this, we established disseminated MM.1S-luc tumors in FCSB mice. As in patients, these mice developed tumors at varying rates and locations. Mice were divided into treatment groups, and response was monitored by BLI (Figure 5A; Figure S4). Of 39 mice, three showed no BLI signal at therapy onset; two were included in the untreated group, and one in the TiO₂-Tf-TC group. Another mouse with a low baseline BLI was assigned to chemo-RaST.

BLI showed significant tumor progression after day 50 in untreated, TiO₂-Tf-TC, bortezomib, and ⁸⁹Zr-daratumumab groups, except one untreated mouse with a low baseline signal (Figure 5A; Figure S4). Quantitative BLI indicated continued tumor growth in untreated, TiO₂-Tf-TC, bortezomib, and ⁸⁹Zr-daratumumab groups (Figure 5B). In contrast, RaST inhibited growth in 60% and constrained growth in 40% by day 50, but relapse occurred by day 60. Chemo-RaST achieved progression-free survival and partial response in 60% and 40% of mice, respectively (Figure 5C). Kaplan-Meier analysis showed that 60% of chemo-RaST mice (complete responders) remained disease-free and survived to study end (day 140), whereas all other mice had succumbed (Figure 5D; Supplementary Table 1).

**Figure 6:**
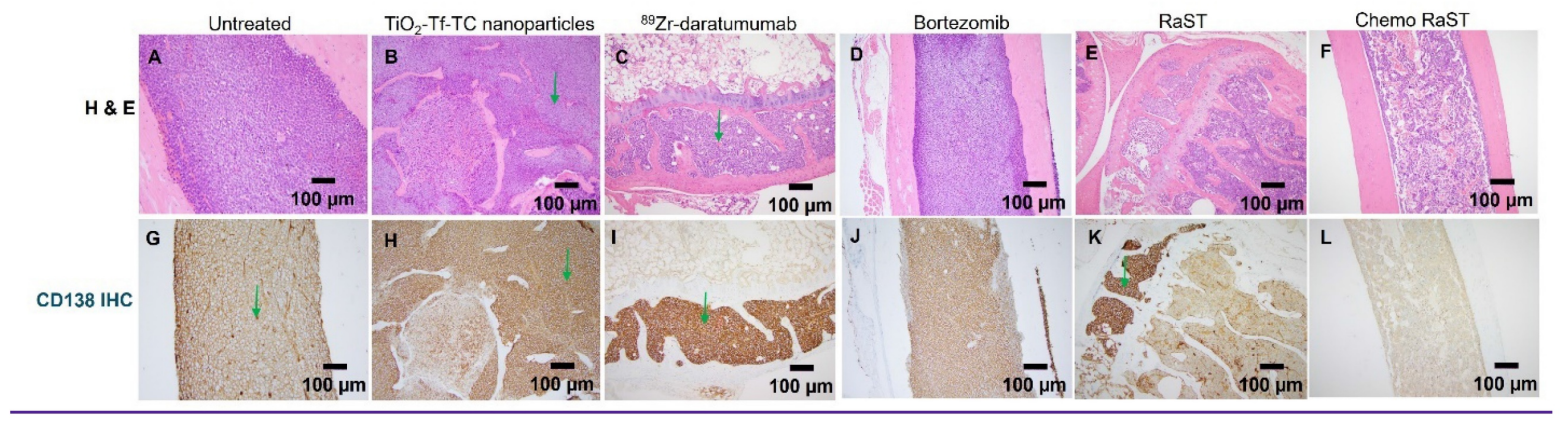
** Histologic comparison 10X (original magnification X 100) of bone marrow involvement by MM cells (green arrows) in untreated and treated cohorts by H&E (A-F), corresponding CD138 (G-L).** (A, G) Untreated cohort with complete marrow replacement by MM (B, H) Treated with TiO2-Tf-TC nanoparticle showing no significant treatment response and persistent extensive MM. (C, I) ^89^Zr-Daratumumab treatment cohorts with partial response and persistent focal MM marrow involvement. (D, J) Bortezomib only cohort with no significant treatment response. (E, K) RaST treatment cohorts with partial response and persistent focal MM marrow involvement. (F, L) Chemo RaST treatment group with complete response, normal bone marrow, and no detectable tumors.

Histology on day 50 leg-bone tissues showed extensive marrow replacement by CD138-positive plasma cells in untreated mice (Figure 6). The ⁸⁹Zr-daratumumab and RaST groups exhibited partial responses, with focal subcortical deposits comprising 5-10% of marrow cellularity and largely normal hematopoiesis. Conversely, chemo-RaST produced complete tumor eradication and normal hematopoiesis.

## Discussion

Most MM patients respond initially but experience remission-relapse cycles that culminate in progression and mortality. Advances in immunotherapies, biologics, and combinations have extended survival but remain inadequate for sustained remission 33. Extending disease-free survival requires effective combinations that address clonal heterogeneity. This study demonstrates that RaST with ⁸⁹Zr-daratumumab and TiO₂-Tf-TC nanophotosensitizers, combined with bortezomib, can achieve durable responses in preclinical MM models.

The choice of radionuclide is critical for success. Prior work with short-lived positron emitters (e.g., ⁶⁸Ga, ¹⁸F) often required multiple high-activity re-injections to sustain inhibition because >95% of positrons/Cerenkov radiation are emitted within ~10 h (for ¹⁸F) and are essentially depleted by 24 h 7, 11, 15, 34. Such brief, high-intensity exposure can allow residual cancer cells time to recover, promote adaptive reprogramming, and increase toxicity risks to surrounding healthy tissues. By contrast, ⁸⁹Zr releases positrons gradually, with ~90% of emissions occurring over ~11 days. The serum half-life of daratumumab in mice (7-8 days) matches the 3.3-day physical half-life of ⁸⁹Zr 24, enabling continuous stimulation of regenerative TiO₂ with only two doses spaced two weeks apart. This avoids frequent re-dosing and efficiently produces ROS with tracer-level radionuclide, significantly improving approaches that rely on short-lived radioisotopes.

High-dose radioimmunoconjugates (e.g., ¹⁷⁷Lu-daratumumab) have yielded strong responses in mice 35, but as with most receptor-mediated delivery, only a fraction of the radioimmunoconjugate internalizes in cancer cells, leaving substantial activity in circulation or at the cell surface and increasing exposure of healthy organs. The prolonged biological half-life of daratumumab at therapeutic doses and the long physical half-life of ⁸⁹Zr can sustain off-target effects before clearance. RaST addresses this by using tracer-level ⁸⁹Zr, comparable to PET doses, which is non-cytotoxic alone. Conceptually, RaST functions like a prodrug, where cytotoxic ROS are produced only when both components co-localize within tissues overexpressing CD71 and CD38 (e.g., MM cells). We administered TiO₂-Tf-TC three days after ⁸⁹Zr-daratumumab. Because daratumumab can deplete CD38 36, subsequent nanoparticle uptake via CD38 could be limited. Our findings show that Tf receptors are expressed on MM.1S-luc cells, providing an orthogonal path to deliver the nanophotosensitizers and circumvent the challenges posed by CD38 depletion.

BLI showed significant inhibition with RaST in the subcutaneous model, but relapse emerged by day 40, revealing limitations of ROS-based monotherapy. Rescue efforts with bortezomib 37 did not inhibit RaST-resistant tumors, suggesting the acquisition of shared resistance mechanisms. Combining bortezomib with RaST yielded complete regression in 60% of mice, consistent with convergence of biological and photophysical ROS generation. Histology supported the advantage of chemo-RaST, showing eradication of CD138-positive cells and restoration of hematopoiesis. Partial responses in RaST-only groups mirror reports where single modalities achieve temporary control without preventing relapse 33. Together, these data support strategies that integrate mechanistically distinct yet synergistic cancer treatments.

Chemo-RaST disrupts cancer cells via multiple death pathways. RaST induces apoptosis, ferroptosis, and necroptosis through ROS generation 7, 11, 12, 38, 39. In parallel, bortezomib triggers proteasome inhibition-mediated ER stress 20, 21, promoting mitochondrial membrane permeabilization and increasing ROS, which culminate in caspase activation and apoptosis. Although the tracer dose of ⁸⁹Zr-daratumumab used here likely contributed minimally to the complete-responder cohort, higher activities could leverage daratumumab's immunotherapeutic mechanisms and the DNA-damaging radiation from ⁸⁹Zr. A key limitation of this study is that the dominant cell-death mechanism underlying chemo-RaST's MM suppression remains unclear. Defining these mechanisms and optimizing component dose and scheduling will be important for optimizing RaST-based combination therapies.

## Conclusion

Chemo-RaST shows therapeutic promise for MM. By engaging both photophysical and mitochondrial ROS-generating agents, the treatment achieved significant tumor regression, reduced relapse, and prolonged progression-free survival in preclinical models. The use of tracer doses of long-lived ⁸⁹Zr for continuous photosensitizer activation highlights the advantages of extended-half-life radionuclides in RaST. This approach is highly translational, supported by prior use of ⁸⁹Zr-daratumumab and bortezomib in patients. Although we used tracer ⁸⁹Zr-daratumumab and a standard bortezomib dose, future work should evaluate dose ranges and refine radionuclide-photosensitizer timing to maximize benefit while minimizing off-target toxicity. Investigating additional agents, including small-molecule inhibitors or immunomodulators, may further enhance chemo-RaST and enable durable, multi-faceted regimens to achieve disease-free remission while maintaining quality of life for MM patients.

## Supplementary Material

Supplementary methods, figures and table.

## Abbreviations

MM: multiple myeloma
NP: nanoparticle
TiO_2_: titanium dioxide
Tf: transferrin
TC: Titanocene
ROS: reactive oxygen species
RaST: radionuclide-stimulated dynamic therapy
chemo-RaST: chemotherapy combined radionuclide stimulated dynamic therapy
TEM: transmission electron microscopy
DLS: dynamic light scattering
BLI: bioluminescence imaging
PET/CT: positron emission tomography/computed tomography
^89^Zr: Zirconium-89
PBS: phosphate buffered saline
iTLC: instant thin layer chromatography
RCP: radiochemical purity
luc: luciferase
FCSB: fox chase SCID beige
ROI: region of interest
SUV: standardized uptake value
IHC: immunohistochemistry
H&E: Hematoxylin and eosin
ICP-OES: Inductively Coupled Plasma-Optical Emission Spectrometer
ICP-MS: inductively coupled plasma mass spectrometry

## References

[1] Cid Ruzafa J, Merinopoulou E, Baggaley RF, Leighton P, Werther W, Felici D, Cox A (2016). Patient population with multiple myeloma and transitions across different lines of therapy in the USA: an epidemiologic model. Pharmacoepidemiol Drug Saf.

[2] Blade J, Fernandez-Llama P, Bosch F, Montoliu J, Lens XM, Montoto S (1998). Renal failure in multiple myeloma: presenting features and predictors of outcome in 94 patients from a single institution. Arch Intern Med.

[3] Kumar SK, Rajkumar SV, Dispenzieri A, Lacy MQ, Hayman SR, Buadi FK (2008). Improved survival in multiple myeloma and the impact of novel therapies. Blood.

[4] Ghai A, Maji D, Cho N, Chanswangphuwana C, Rettig M, Shen D (2018). Preclinical Development of CD38-Targeted [(^89^)Zr]Zr-DFO-Daratumumab for Imaging Multiple Myeloma. J Nucl Med.

[5] Usmani SZ, Rodriguez-Otero P, Bhutani M, Mateos MV, Miguel JS (2015). Defining and treating high-risk multiple myeloma. Leukemia.

[6] Gay F, Engelhardt M, Terpos E, Wäsch R, Giaccone L, Auner HW (2018). From transplant to novel cellular therapies in multiple myeloma: European Myeloma Network guidelines and future perspectives. Haematologica.

[7] Kotagiri N, Sudlow GP, Akers WJ, Achilefu S (2015). Breaking the depth dependency of phototherapy with Cerenkov radiation and low-radiance-responsive nanophotosensitizers. Nat Nanotechnol.

[8] Lane DD, Black KCL, Raliya R, Reed N, Kotagiri N, Gilson R (2020). Effects of core titanium crystal dimension and crystal phase on ROS generation and tumour accumulation of transferrin coated titanium dioxide nanoaggregates. RSC Adv.

[9] Shaffer TM, Pratt EC, Grimm J (2017). Utilizing the power of Cerenkov light with nanotechnology. Nat Nanotechnol.

[10] Liu H, Wang Q, Guo J, Feng K, Ruan Y, Zhang Z (2023). Prodrug-based strategy with a two-in-one liposome for Cerenkov-induced photodynamic therapy and chemotherapy. J Control Release.

[11] Kotagiri N, Cooper ML, Rettig M, Egbulefu C, Prior J, Cui G (2018). Radionuclides transform chemotherapeutics into phototherapeutics for precise treatment of disseminated cancer. Nat Commun.

[12] Zheleznyak A, Mixdorf M, Marsala L, Prior J, Yang X, Cui G (2021). Orthogonal targeting of osteoclasts and myeloma cells for radionuclide stimulated dynamic therapy induces multidimensional cell death pathways. Theranostics.

[13] Ruggiero A, Holland JP, Lewis JP, Grimm J (2010). Cerenkov luminescence imaging of medical isotopes. J Nucl Med.

[14] Zhang Y, Hao Y, Chen S, Xu M (2020). Photodynamic Therapy of Cancers With Internal Light Sources: Chemiluminescence, Bioluminescence, and Cerenkov Radiation. Front Chem.

[15] Duan D, Liu H, Xu Y, Han Y, Xu M, Zhang Z (2018). Activating TiO2 Nanoparticles: Gallium-68 Serves as a High-Yield Photon Emitter for Cerenkov-Induced Photodynamic Therapy. ACS Appl Mater Interfaces.

[16] Zhang Y, Hong H, Cai W (2011). PET tracers based on Zirconium-89. Curr Radiopharm.

[17] Caserta E, Chea J, Minnix M, Poku EK, Viola D, Vonderfecht S (2018). Copper 64-labeled daratumumab as a PET/CT imaging tracer for multiple myeloma. Blood.

[18] Linsebigler AL, Lu G, Yates Jr JT (1995). Photocatalysis on TiO2 surfaces: principles, mechanisms, and selected results. Chem Rev.

[19] Gatter KC, Brown G, Trowbridge IS, Woolston RE, Mason DY (1983). Transferrin receptors in human tissues: their distribution and possible clinical relevance. J Clin Pathol.

[20] Mateos MV, Oriol A, Martinez-Lopez J, Gutierrez N, Teruel AI, de Paz R (2010). Bortezomib, melphalan, and prednisone versus bortezomib, thalidomide, and prednisone as induction therapy followed by maintenance treatment with bortezomib and thalidomide versus bortezomib and prednisone in elderly patients with untreated multiple myeloma: a randomised trial. Lancet Oncol.

[21] Kouroukis TC, Baldassarre FG, Haynes AE, Imrie K, Reece DE, Cheung MC, Bortezomib in multiple myeloma (2014). systematic review and clinical considerations. Curr Oncol.

[22] Ishii T, Seike T, Nakashima T, Juliger S, Maharaj L, Soga S (2012). Anti-tumor activity against multiple myeloma by combination of KW-2478, an Hsp90 inhibitor, with bortezomib. Blood Cancer J.

[23] Boccadoro M, Morgan G, Cavenagh J (2005). Preclinical evaluation of the proteasome inhibitor bortezomib in cancer therapy. Cancer Cell Int.

[24] Zeglis BM, Lewis JS (2015). The bioconjugation and radiosynthesis of 89Zr-DFO-labeled antibodies. J Vis Exp.

[25] Greenstein S, Krett NL, Kurosawa Y, Ma C, Chauhan D, Hideshima T (2003). Characterization of the MM.1 human multiple myeloma (MM) cell lines: a model system to elucidate the characteristics, behavior, and signaling of steroid-sensitive and -resistant MM cells. Exp Hematol.

[26] Lokhorst HM, Plesner T, Laubach JP, Nahi H, Gimsing P, Hansson M (2015). Targeting CD38 with Daratumumab Monotherapy in Multiple Myeloma. N Engl J Med.

[27] Blade J, Beksac M, Caers J, Jurczyszyn A, von Lilienfeld-Toal M, Moreau P (2022). Extramedullary disease in multiple myeloma: a systematic literature review. Blood Cancer J.

[28] Lü S, Wang J (2013). The resistance mechanisms of proteasome inhibitor bortezomib. Biomark Res.

[29] Pérez-Galán P, Roué GI, Villamor N, Montserrat E, Campo E, Colomer D (2006). The proteasome inhibitor bortezomib induces apoptosis in mantle-cell lymphoma through generation of ROS and Noxa activation independent of p53 status. Blood.

[30] Sonneveld P (2017). Management of multiple myeloma in the relapsed/refractory patient. Hematology Am Soc Hematol Educ Program.

[31] Song IS, Kim HK, Lee SR, Jeong SH, Kim N, Ko KS (2013). Mitochondrial modulation decreases the bortezomib-resistance in multiple myeloma cells. Int J Cancer.

[32] Song IS, Jeong YJ, Jeong SH, Heo HJ, Kim HK, Lee SR (2013). Combination treatment with 2-methoxyestradiol overcomes bortezomib resistance of multiple myeloma cells. Exp Mol Med.

[33] Dima D, Jiang D, Singh DJ, Hasipek M, Shah HS, Ullah F (2022). Multiple Myeloma Therapy: Emerging Trends and Challenges. Cancers (Basel).

[34] Nakamura Y, Nagaya T, Sato K, Okuyama S, Ogata F, Wong K (2017). Cerenkov Radiation-Induced Photoimmunotherapy with ^18^F-FDG. J Nucl Med.

[35] Kang L, Li C, Rosenkrans ZT, Huo N, Chen Z, Ehlerding EB (2021). CD38-Targeted Theranostics of Lymphoma with (89)Zr/(177)Lu-Labeled Daratumumab. Adv Sci (Weinh).

[36] Saltarella I, Desantis V, Melaccio A, Solimando AG, Lamanuzzi A, Ria R (2020). Mechanisms of Resistance to Anti-CD38 Daratumumab in Multiple Myeloma. Cells.

[37] Painuly U, Kumar S (2013). Efficacy of bortezomib as first-line treatment for patients with multiple myeloma. Clin Med Insights Oncol.

[38] Tang R, Zheleznyak A, Mixdorf M, Ghai A, Prior J, Black KCL (2020). Osteotropic Radiolabeled Nanophotosensitizer for Imaging and Treating Multiple Myeloma. ACS Nano.

[39] Egbulefu C, Black K, Su X, Karmakar P, Habimana-Griffin L, Sudlow G (2024). Induction of complementary immunogenic necroptosis and apoptosis cell death pathways inhibits cancer metastasis and relapse. Res Sq.

