# Supplementary material for: Chemo-RaST with bortezomib inhibits multiple myeloma relapse
Source: Theranostics. 2025 Sep 8;15(18):9911–21. doi: 10.7150/thno.111508 (PMC12486394; doi:10.7150/thno.111508)
Supplement: Supplementary file 1 — Supplementary methods, figures and table. [file thnov15p9911s1.pdf]

Combined Bortezomib and Cerenkov radiating ^{89}Zr-daratumumab-mediated dynamic therapy disrupts frequent multiple myeloma relapse

Anchal Ghai[1,2#], Alexander Zheleznyak[1], Christopher Egbulefu[1,2#], Nicole Blasi[2], Kvar Black[1], Rui Tang[1], Mathew Cooper[3], Kiran Vij[3, 4], Ravi Vij[3], John DiPersio[3], Monica Shokeen[1,3,5], Samuel Achilefu[1,2#, 3,5, 6*]

Affiliations:

[1]Department of Radiology, Washington University School of Medicine, St. Louis, Missouri, USA

[2]Department of Biomedical Engineering, UT Southwestern Medical Center, Dallas, Texas, USA

[3]Department of Medicine, Washington University School of Medicine, Missouri, USA

[4]Department of Pathology and Immunology, Washington University School of Medicine, Missouri, USA

[5]Department of Biomedical Engineering, Washington University in St. Louis, Missouri, USA

[6]Department of Biochemistry and Molecular Biophysics, Washington University School of Medicine, Missouri, USA

* Samuel Achilefu, Department of Biomedical Engineering, UT Southwestern Medical Center, 2336 Inwood Road, Dallas, TX, 75390-9397, USA.

Email: Samuel.achilefu@utsouthwestern.edu

[#] Current affiliation

Table of Contents

Supplemental methods…………………………………………………………………………….. 2-3

Figure S1………………………………………………………………………………............... 4

Figure S2……………………………………………………………………………………….... 5

Figure S3……………………………………………………………………………………….... 6

Figure S4……………………………………………………………………………………….... 7

Figure S5……………………………………………………………………………………….... 8

Figure S6……………………………………………………………………………………….... 9

Table 1…………………………………………………………………………………………...... 10

Supplemental methods:

Quality control of ^{89}Zr-daratumumab

Daratumumab-DFO was radiolabeled with ^{89}Zr-oxalate and the radiochemical purity was evaluated by radio thin-layer chromatography using 50mM diethylenetriaminepentaacetic acid as the mobile solvent. The chromatogram shows ^{89}Zr labeled antibody stays at the origin whereas free ^{89}Zr moves with the solvent front (Figure S1).

Bioluminescent imaging (BLI)

The tumor progression *in vivo* was monitored weekly by BLI in MM.1S subcutaneous tumor bearing and disseminated tumor bearing mouse models using IVIS Lumina (PerkinElmer, Waltham, MA, USA; Living Image 3.2, 1-300 sec exposures, binning 2-8, field of view (FOV) 12.5 cm). Mice were injected with 150mg/kg D luciferin in PBS intraperitoneal and imaged 10 min post injection under isoflurane anesthesia (2% vaporized in O_2). Regions of interest (ROIs) were drawn on the tumors and over the entire dorsal/ventral side of the mouse in the vase of subcutaneous and disseminated tumor bearing mice, respectively. The total flux (photons/sec) was measured from these ROIs using Living Image 2.6 software.

In vitro mitochondrial ROS detection assay

Mitochondrial ROS production in MM.1S cells was evaluated using the Mitochondrial ROS detection kit (Cayman Chemicals). Briefly, 0.2e6 cells/well were plated in a U-bottom 96 well plate in 100 µL of complete RPMI culture media. Cells were treated with different concentrations of bortezomib (1.25 nM- 20 nM) for 24 h at 37°C, 5% CO_2. After treatment, cells were pelleted by centrifugation at 400g for 1-2 minutes, washed once with the cell-based assay buffer (provided in the kit), and resuspended in 100 µL of mitochondrial ROS detection reagent (prepared fresh according to the manual instructions). Cells were incubated with the reagent in dark for 20 min at

37°C, then washed thrice with assay buffer to remove unbound dye. Untreated cells (negative control) and cells treated with Antimycin A (10 µM; for 1 h at 37°C; positive control) were included on the plate. After a final resuspension in 100 µL of cell-based assay buffer, fluorescence was measured using a microplate reader at excitation/emission = 510/580 nm. Background fluorescence was subtracted, and all measurements were normalized to cell number. Results were expressed as %ROS and compared across treated, untreated, and positive control groups.

SUPPLEMENTARY FIGURES:

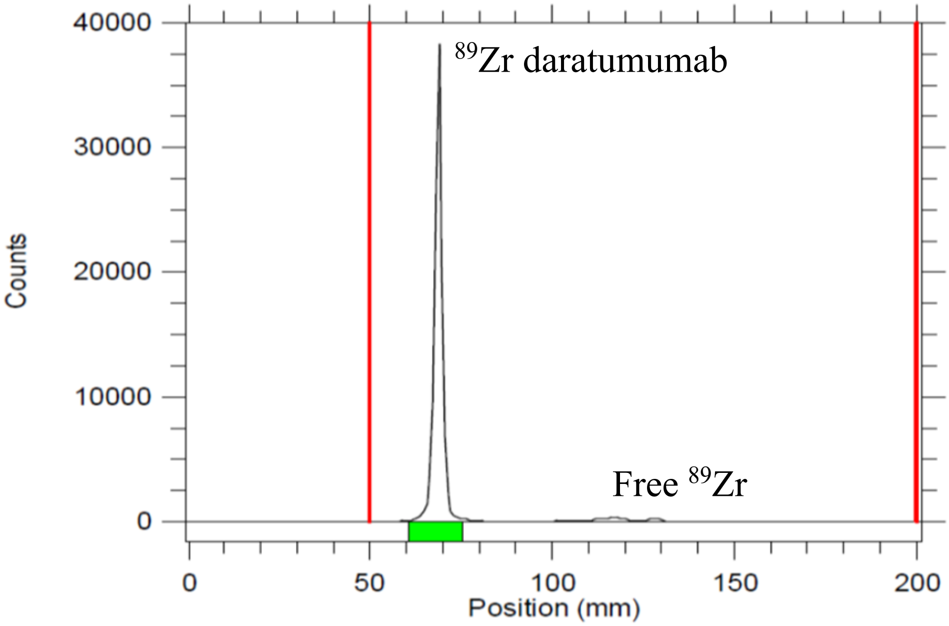

Figure S1. radio-TLC scan of purified labeled ^{89}Zr-DFO-daratumumab conjugate.

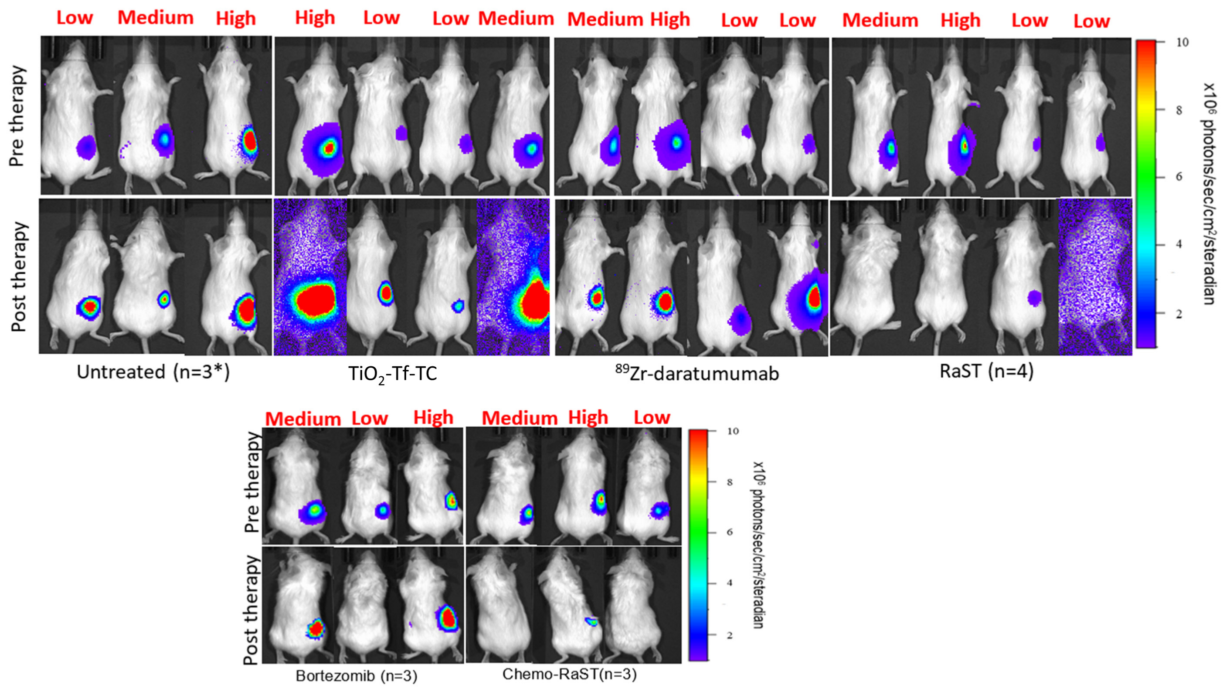

Figure S2. BLI images for MM.1S subcutaneous tumor-bearing animals showing tumor progression in different treatment groups pre- (day 21) and post-therapy (day 38). The mice were staggered equally in all the groups based on different tumor burdens (low, medium, and high). Different initial tumor burdens are highlighted in red. The color bar is set to a lower scale; therefore, we can see the BLI signal in mice with low tumor burden too. * We started with n=4, one mouse died after the injection.

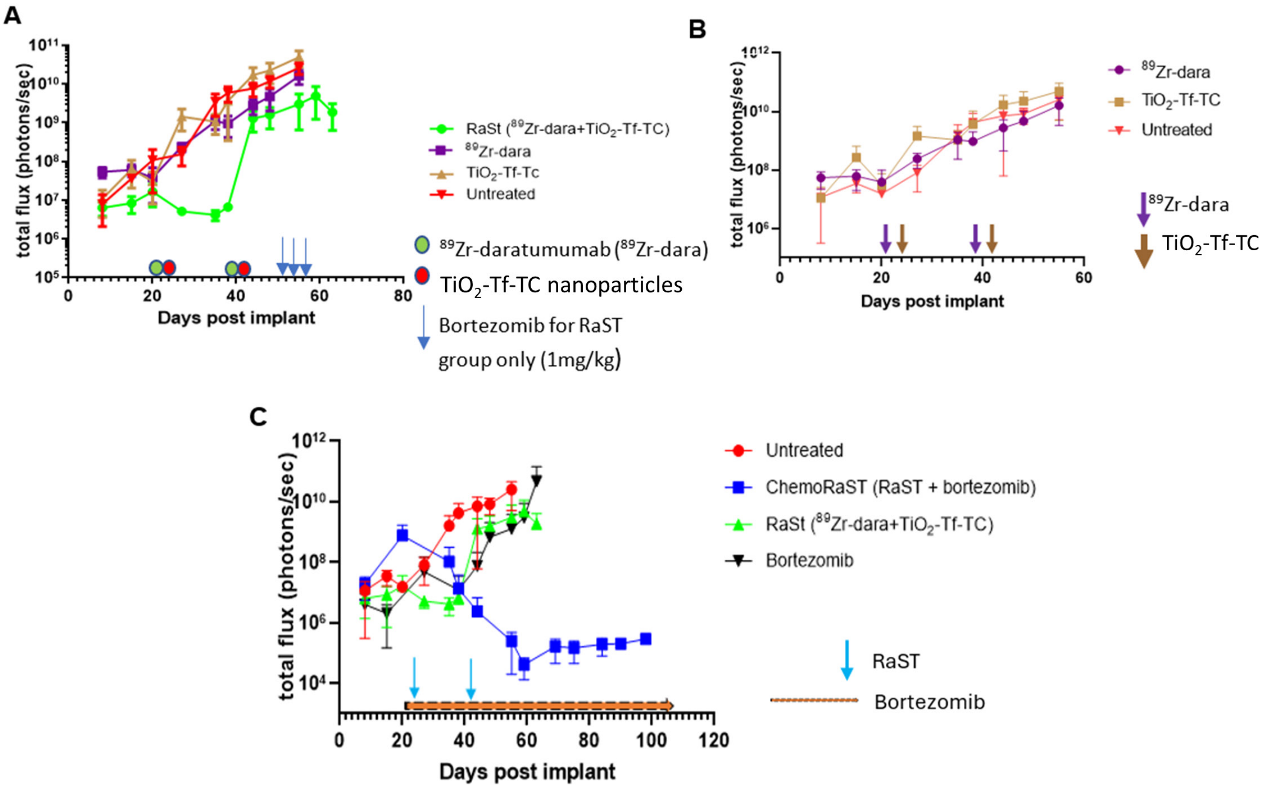

Figure S3. A. Bioluminescence signal intensity was quantified for MM.1S-luc subcutaneous tumor-bearing animals of each group (n=4/group) plotted and is shown as a function of the number of days after transplantation. ^{89}Zr-daratumumab was injected at day 21 and day 39 followed by TiO_2-Tf-TC nanoparticle injections at day 24 and day 42 for the RaST group. Bortezomib (1mg/kg) therapy started on day 51, twice a week. **B.** BLI was quantified for MM.1S-luc subcutaneous tumor-bearing mice (n=4/group), and the BLI signal of the untreated group was compared with mice treated with ^{89}Zr-daratumumab only, and TiO_2-Tf-TC nanoparticles only (plotted and is shown as a function of the number of days after transplantation). **C.** BLI was quantified for MM.1S-luc subcutaneous tumor-bearing mice of each group plotted and is shown as a function of the number of days after transplantation. Bortezomib (1mg/kg) therapy was started on day 21, twice a week for mice treated with RaST in combination with bortezomib.

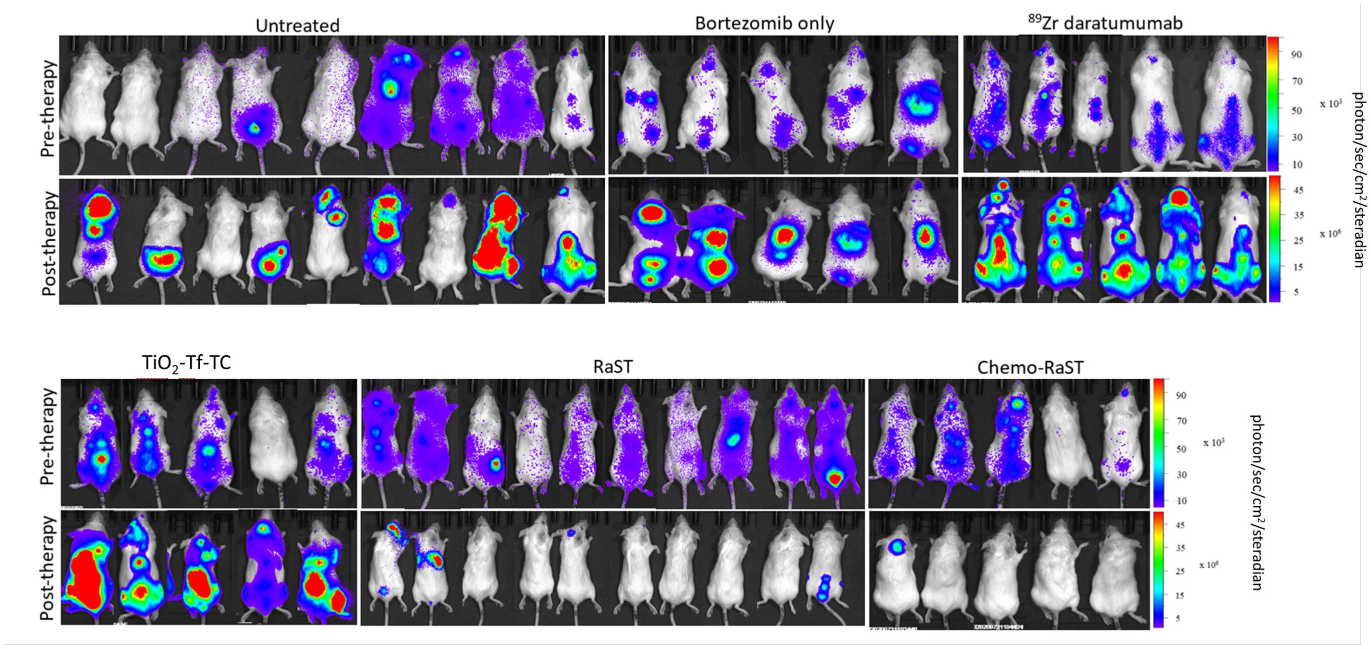

Figure S4. BLI images for MM.1S-luc disseminated tumor-bearing animals showing tumor progression in different treatment groups pre (day 21) and post (day 50)- therapy. The mice were staggered equally in all the groups based on different tumor burdens (low, medium, and high). The color bar is set to a lower scale; therefore, we can also see the BLI signal in mice with low tumor burden.

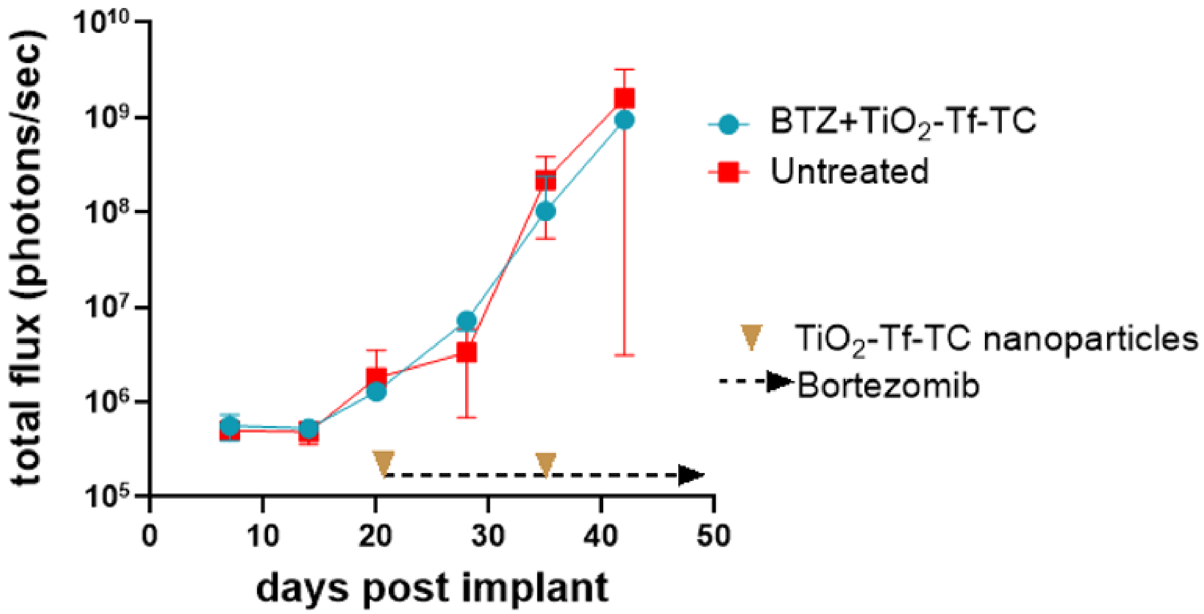

Figure S5. Bioluminescence signal intensity in MM.1S-luc disseminated tumor–bearing mice (n = 3 per group) plotted versus days post-transplantation. TiO_2-Tf-TC nanoparticles were administered on days 21 and 39. Bortezomib treatment (1 mg/kg, intraperitoneal injection) began on day 21 and was given twice weekly thereafter.

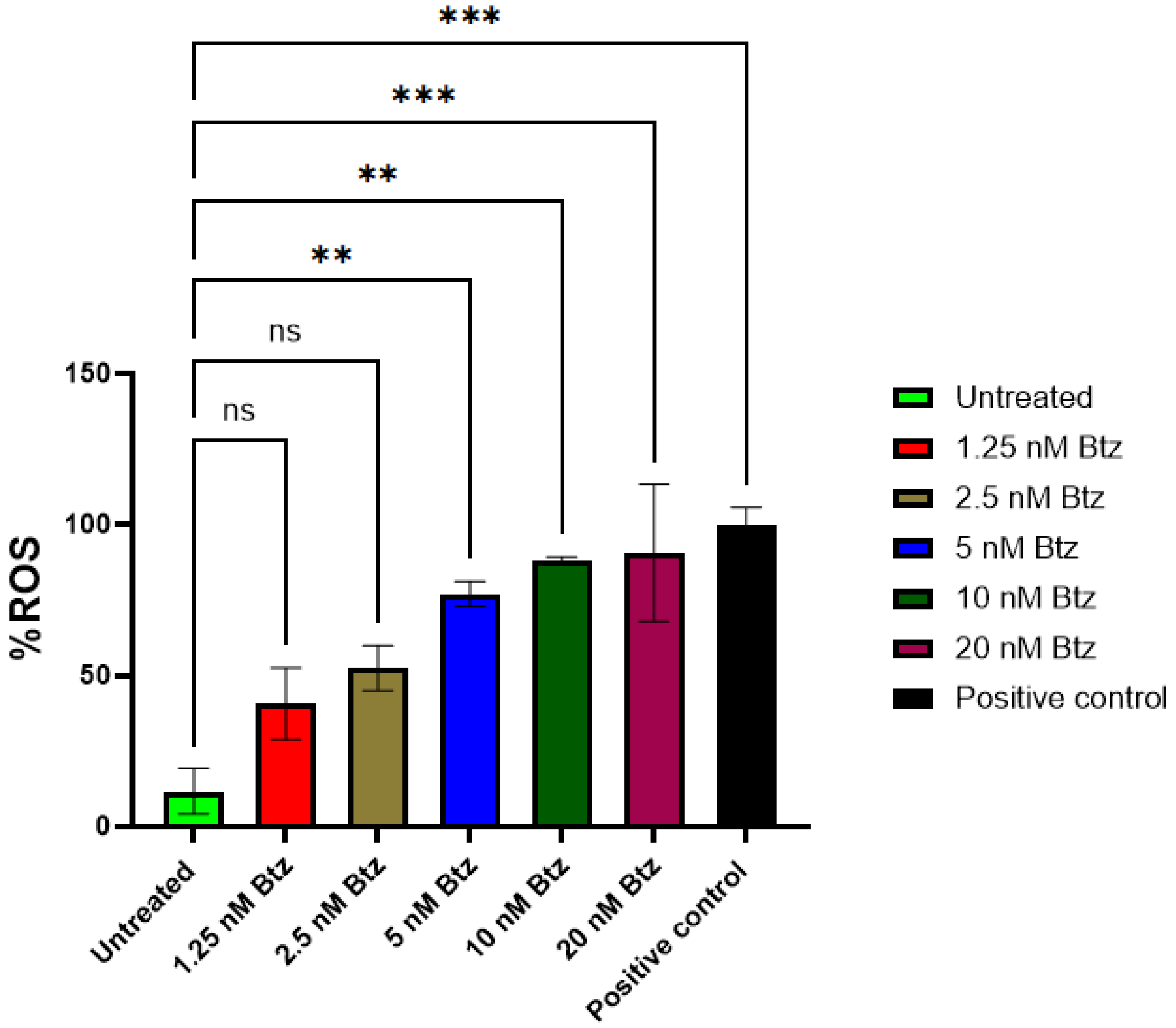

Figure S6. Bortezomib induces mitochondrial ROS in a dose-dependent manner. MM.1S cells were treated with increasing concentrations of bortezomib (1.25-20 nM) for 24 h, and mitochondrial ROS levels were assessed using a fluorescent ROS detection assay. Results are expressed as percent ROS relative to a positive control set to 100%. Bars show mean ± SEM ($n = 3$). Statistical significance was determined by one-way ANOVA: $^{**}p < 0.01$, $^{***}p < 0.001$; ns, not significant.

Table 1 Kaplan-Meir survival curve analysis of FCSB mice with disseminated myeloma disease with different treatments

**Treatment**	**N (sample size)**	**% survival on day 140**
Untreated	9	0
TiO_2-Tf-TC nanoparticles	5	0
^{89}Zr-daratumumab	5	0
Bortezomib	5	0
RaST	10	0
Chemo-RaST	5	60
-Partial responders	2	0
-complete responders	3	100
